# Neuropilin-2 Is Associated With Increased Hepatoblastoma Cell Viability and Motility

**DOI:** 10.3389/fped.2021.660482

**Published:** 2021-06-22

**Authors:** Katja Eloranta, Ruth Nousiainen, Stefano Cairo, Mikko P. Pakarinen, David B. Wilson, Marjut Pihlajoki, Markku Heikinheimo

**Affiliations:** ^1^Pediatric Research Center, Children's Hospital, Helsinki University Hospital, University of Helsinki, Helsinki, Finland; ^2^XenTech, Evry, France; ^3^Istituto di Ricerca Pediatrica, Padova, Italy; ^4^Pediatric Surgery, and Pediatric Liver and Gut Research Group, Children's Hospital, Helsinki University Hospital, University of Helsinki, Helsinki, Finland; ^5^Department of Pediatrics, Washington University School of Medicine, St. Louis Children's Hospital, St. Louis, MO, United States; ^6^Department of Developmental Biology, Washington University School of Medicine, St. Louis, MO, United States

**Keywords:** neuropilin, hepatoblastoma, pediatric cancer, cell viability, migration, liver

## Abstract

The neuropilins NRP1 and NRP2 are multifunctional glycoproteins that have been implicated in several cancer-related processes including cell survival, migration, and invasion in various tumor types. Here, we examine the role of neuropilins in hepatoblastoma (HB), the most common pediatric liver malignancy. Using a combination of immunohistochemistry, RNA analysis and western blotting, we observed high level expression of *NRP1* and *NRP2* in 19 of 20 HB specimens and in a majority of human HB cell lines (HUH6 and five cell lines established from patient-derived xenografts) studied but not in normal hepatocytes. Silencing of *NRP2* expression in HUH6 and HB-282 HB cells resulted in decreased cell viability, impaired cytoskeleton remodeling, and reduced cell motility, suggesting that NRP2 contributes to the malignant phenotype. We propose that neuropilins warrant further investigation as biomarkers of HB and potential therapeutic targets.

## Introduction

Hepatoblastoma (HB) is the most common primary liver malignancy in the pediatric population with an incidence of 1.9 cases per million (1, 2). Preterm birth, low birthweight, and certain genetic conditions such as Beckwith-Wiedemann syndrome and Familial Adenomatous Polyposis are associated with increased risk of HB. In most HB cases, however, the etiology of the disease remains unidentified (3, 4). HB histology resembles embryonal or fetal liver, and low differentiation stage associates with poor prognosis (5). The molecular pathways involved in the pathogenesis of HB are not fully understood, although aberrant activation of WNT/β-catenin signaling is present in the majority of these tumors (6, 7).

The neuropilin (NRP) family contains two single-passing transmembrane glycoproteins, neuropilin-1 (NRP1) and neuropilin-2 (NRP2), sharing 44% amino acid sequence homology (8). NRPs interact with plethora of cancer associated pathways encompassing signaling mediated by vascular endothelial growth factors, semaphorins, transforming growth factor beta, hepatocyte growth factor, platelet derived growth factors, and integrins (9–16). NRPs were originally documented as regulators for neurogenesis, angiogenesis and lymphangiogenesis, but there is growing evidence that these glycoproteins are involved in the initiation and progression of various malignancies including hepatocellular carcinoma, pancreatic adenocarcinoma, colorectal adenocarcinoma, breast cancer, and non-small cell lung cancer (17–23). More precisely, overexpression of NRPs has been linked to increased cancer cell viability, motility and invasiveness, as well as resistance to chemotherapy (24–27).

In normal liver, neither NRP1 nor NRP2 is expressed in hepatocytes, whereas NRP1 immunoreactivity is evident in hepatic stellate cells (HSCs) and liver sinusoidal endothelial cells (LSECs) (28, 29).

Here, we characterize the expression patterns of NRP1 and NRP2 in human HB specimens and cell lines. Additionally, we examine the functional consequences of *NRP2* gene silencing in HB cells.

## Patients and Methods

### Patient Samples

Archival formalin-fixed paraffin-embedded (FFPE) HB patient samples (*n* = 20) and normal liver control (NL, *n* = 4) samples were obtained from the Helsinki Biobank at Helsinki University Hospital. The HB samples were originally collected at the time of surgical treatment, whereas the NL samples were from liver transplantation donors. This study was approved by Helsinki University Hospital institutional ethical committee (HUS/3319/2018) and conducted in accordance with Finnish bylaws. Informed consent was obtained when samples were deposited to the Helsinki Biobank.

### Immunohistochemistry

FFPE samples were cut to 5-μm sections and deparaffinized with xylene. For antigen epitope unmasking, samples were treated with antigen target retrieval solution (pH 9; Dako, Glostrup, Denmark) for 30 min at +98°C. Next, endogenous peroxidase activity was blocked with 3% hydrogen peroxidase and non-specific binding was averted with 0.4% casein (both solutions from Novolink Polymer Detection System Kit; Leica, Newcastle, UK). Primary antibody incubations were performed either at +4°C for overnight (NRP2 at dilution 1:2,000; sc-13117, Santa Cruz Biotechnology, Santa Cruz, CA, USA) or at room temperature for 1 h (NRP1 at dilution 1:3,000; ab81321, Abcam, Cambridge, MA). Antibody binding was visualized with polymerized reporter enzyme staining system (Novolink Polymer Detection System Kit). Positive LSEC staining was used as an internal control for both antibodies. Immunoreactivity was scored based on intensity (negative, low/intermediate, or high) by two separate observers. Imaging was performed using 3DHISTECH Panoramic 250 FLASH II digital slide scanner at Genome Biology Unit (Research Programs Unit, Faculty of Medicine, University of Helsinki Biocenter, Helsinki, Finland).

### Cell Lines and Primary Hepatocyte Culture

Human HB cell line HUH6 was obtained from Japanese Collection of Research Bioresources Cell Bank (Osaka, Japan). HB cell lines established from patient-derived xenografts (PDX; HB-282, HB-295, HB-279, HB-284, and HB-243) were provided by XenTech (Evry, France) (30). Primary hepatocytes from a 4-year-old Caucasian male donor with non-liver related cause of death were purchased from Lonza (Basel, Switzerland) and cultured as instructed. HUH6 cells were maintained with Dulbecco's modified Eagle's medium (DMEM)-glutaMAX supplemented with 10 % fetal bovine serum (FBS), 100 U/ml penicillin, and 100 μg/ml streptomycin sulfate (all from Gibco). PDX-derived cell lines were cultured in Advanced DMEM/F12 (Gibco) supplemented with 8 % FBS, 2 mM glutaMAX, 100 U/ml penicillin, and 100 μg/ml streptomycin sulfate, and 20 μM rock kinase inhibitor Y-27632 (S1049; SelleckChem, Houston, TX, USA). All cell lines were regularly tested to confirm absence of mycoplasma with PCR-based method (PromoCell, Heidelberg, Germany).

### RNA Sequencing Data Analysis

To evaluate *NRP1* and *NRP2* mRNA expression, RNA sequencing datasets from previously published studies were downloaded from GEO database of National Center for Biotechnology Information (https://www.ncbi.nlm.nih.gov/geo/) (31) or EGA European Genome-phenome Archive (https://ega-archive.org/). Accession numbers were as following: GSE83518 (HUH6), EGAS00001004827/EGAD00001006621 (HB-282, HB-295, HB-279, HB-284, HB-243), and GSE140520 (adult primary hepatocytes) (32–34). Data was processed utilizing Chipster software (https://chipster.rahtiapp.fi/) (35). Reads were preprocessed using Trimmomatic and then aligned to human reference genome Homo_sapiens.GRCh38.95 using HISAT2 tool. Reads per genes were counted employing HTSeq. Differential expression analysis was conducted with the edgeR-package. Differentially expressed genes were then filtered using cut-off criteria adjusted *p*-value < 0.05 and |logFC|≥1.0.

### NRP2 Silencing

*NRP2* expression was inhibited in HUH6 and HB-282 cells via small interfering RNA (siRNA) transfection. Briefly, adherent HUH6 cells were exposed to 100 nM and HB-282 cells to 25 nM of *NRP2* ON-TARGETplus SMARTpool siRNA or ON-TARGETplus non-targeting (NT) control siRNA (both from Horizon Discovery, Cambridge, UK). Lipofectamine RNAiMAX reagent (Invitrogen, Carlsbad, CA, USA) was used to deliver siRNAs into the HUH6 cells and Dharmafect 4 (Horizon Discovery) was utilized for HB-282 cells. Knockdown efficacy was evaluated at mRNA and protein level 72 h after initiation of transfection. Transfection efficacy was assessed with siGLO Green transfection indicator (Horizon Discovery). A detailed transfection protocol for HUH6 cells is described elsewhere (36), and HB-282 cells were transfected following manufacturer's instructions for Dharmafect.

### RNA and Protein Extraction

A NucleoSpin RNA/Protein extraction kit was utilized for total RNA and protein extractions (Macherey-Nagel, Düren, Germany) following the manufacturer's instructions.

### Quantitative Real-Time Polymerase Chain Reaction

Reverse transcription was carried out using the Reverse Transcriptase Core Kit (Eurogentec, Seraing, Belgium). Quantitative polymerase chain reaction (qPCR) was performed using MESA GREEN qPCR MasterMix Plus SYBR assay (Eurogentec). The geometric mean of *GAPDH* and *PPIG* expression served as a reference. Primer sequences were designed as follows: *GAPDH* GGTCATCCATGACAACTTTGG (forward), CCATCCACAGTCTTCTGGGT (reverse); *NRP2* CTGTGGGTCATCCGTGAGGAC (forward), ATGGGTTCCATGCAGTTCTCCAG (reverse); *PPIG* CAATGGCCAACAGAGGGAAG (forward), CCAAAAACAACATGATGCCCA (reverse).

### Western Blotting

Equal amounts of protein were subjected to electrophoresis using Mini-Protean TGX Stain-Free Gels (Bio-Rad, Hercules, CA, USA) and then transferred onto polyvinyl fluoride membrane. Blocking was performed with 5% non-fat milk in Tris-Buffered Saline. Primary antibody incubations were carried out at +4°C for overnight (NRP1 at dilution 1:1,500, ab81321, Abcam; NRP2 at dilution 1:800, sc-13117, Santa Cruz). Secondary antibody incubation was performed at room temperature for 1 h (1:10,000; #115-005-062 or #111-035-144, Jackson ImmunoResearch, West Grove, PA, USA). Protein bands were illuminated utilizing the Enhanced Chemiluminescence detection kit (Amersham ECL reagent; GE Healthcare, Barrington, IL). Quantification was performed with Image Lab Software 6.0 (Bio-Rad). NRP1 and NRP2 band intensities were normalized to amount of total protein in corresponding lane (Supplementary Figure 1) utilizing stain-free technology (37).

### Viability Measurements

Cells were seeded into 96-well plates and transfected with *NRP2* or NT siRNA. Cell viability was measured utilizing clonogenic assay and ATPlite assay (PerkinElmer, Waltham, MA, USA) at 72 h post-transfection. For clonogenic assay, transfected cells were seeded at low densities into 6-well plates. After 72 h, cells were fixed with 4% paraformaldehyde, permeabilized with 100% methanol, and consequently stained with crystal violet solution. Images were collected with Bio-Rad ChemiDoc XRS+ Imaging System. The number of colonies were quantified with ImageJ software. ATPlite assay was performed following the manufacturer's instructions, and luminescence was measured with an Enspire Multimode Plate Reader (PerkinElmer).

### Migration Assay

Cell migration was evaluated utilizing transwell migration inserts (8 μm pore size; Merck Millipore, Darmstadt, Germany). The bottom of each insert was pre-coated with collagen I (0.1 mg/ml; Sigma Aldrich, St. Louis, MO, USA), and the inserts were placed into the 24-well plates containing normal cell culture medium. *NRP2* or NT siRNA transfected cells (at density of 50 × 10^3^/insert for HUH6 cells and 20 × 10^3^/insert for HB-282 cells) were seeded to upper side of membrane in serum-free medium. After 40 h, cells were fixed with 4% paraformaldehyde, permeabilized with 100% methanol and stained with crystal violet solution. Non-migrated cells were removed from upper side of membrane with cotton swab. In each insert, images were captured from five randomly chosen fields with Eclipse TS100 microscope supplemented with DS-Fi1 digital imaging system (magnification 10x; Nikon, Tokyo, Japan). The number of migrated cells was assessed with ImageJ software.

### Immunofluorescence Staining

*NRP2* or NT siRNA transfected HUH6 cells were grown in 2-well chamber slides coated with collagen I for 72 h. Fixation was performed with 4% paraformaldehyde. Following, 0.1% Triton-X was used for permeabilization. Non-specific binding was blocked with UltraVision Protein Block solution (Thermo Scientific, Fremont, CA, USA). F-actin staining was carried out with phalloidin-FITC (at dilution 1:500; P5282, Sigma Aldrich) at room temperature for 1 h. Images were captured with Zeiss Axio Imager M2 (objective: EC Plan Neofluar 40 × /0.75 Ph 2 M27) (Carl-Zeiss, Oberkochen, Germany).

### Statistical Analysis

For qPCR, protein analysis, viability assays, and migration studies, three independent experiments were conducted. Statistical significance was assessed with Student's *t*-test utilizing JMP Software (JMP Pro; version 15.1.0, SAS Institute Inc.). A *p*-value < 0.05 was considered as statistically significant. RNA-sequencing data was analyzed with edgeR-package and significance level was set at adjusted *p*-value < 0.05.

## Results

### NRPs Are Highly Expressed in HB Tissue and Cell Lines

**Figure 1:**
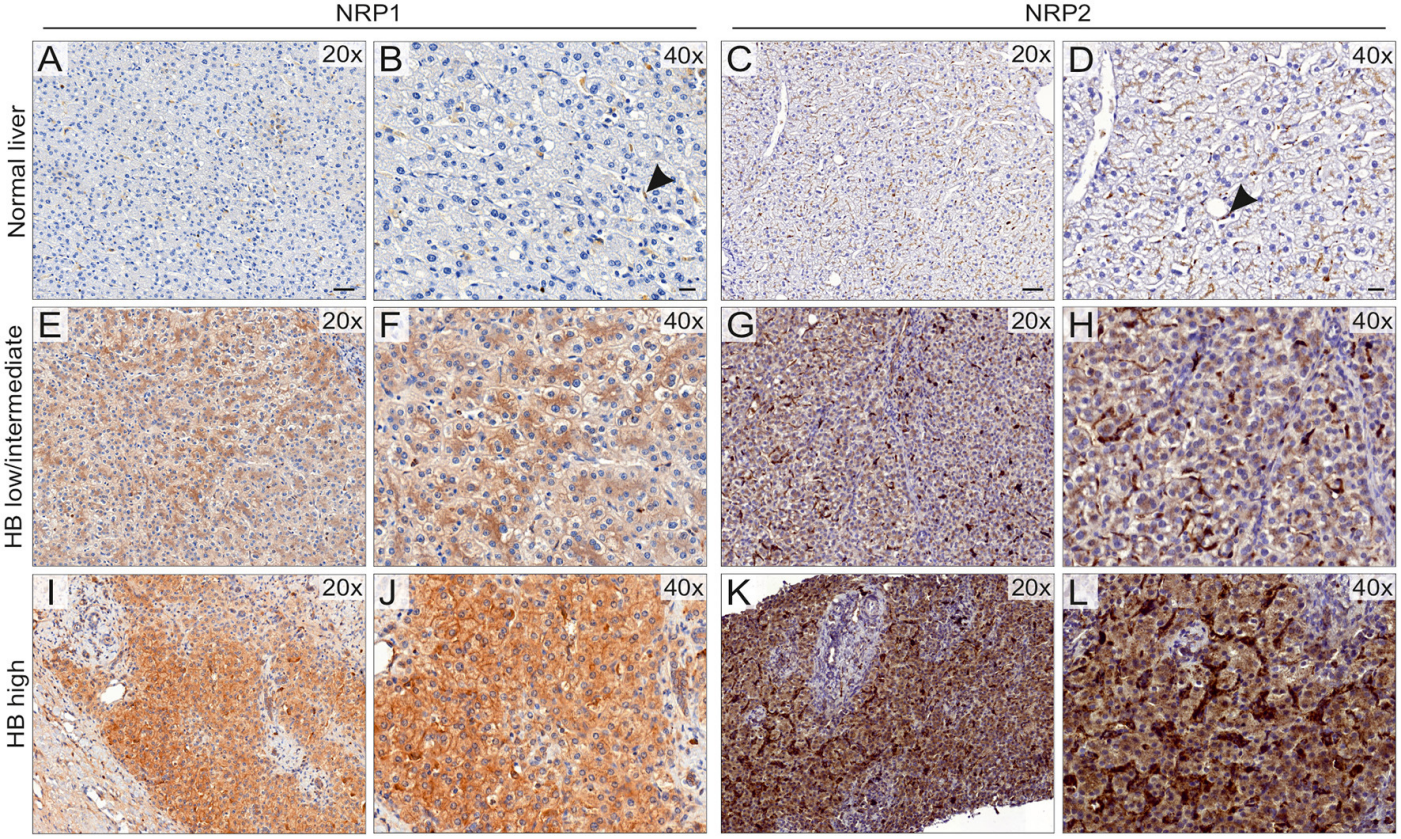
NRP1 and NRP2 expression in HB patient samples and normal liver. NRP1 and NRP2 expression was limited to LSCEs (arrowhead) in normal liver (*n* = 4) **(A–D)**. HB tumor cells demonstrated low/intermediate (10/20) **(E,F)** or high (10/20) **(I,J)** NRP1 immunoreactivity localized to the cell membrane and cytoplasm. NRP2 expression **(G,H)** was low/intermediate in 11/20 HBs. High NRP2 **(K,L)** immunoreactivity was observed in 9/20 of HBs. NRP2 was mainly detected in the cytoplasm with lesser amounts in membranes **(G,H,K,L)**. Scale bars: 50 μm **(A,C)**, 20 μm **(B,D)**.

We assessed NRP1 and NRP2 expression in FFPE samples collected from 20 HB patients treated at Helsinki University Hospital between January 1, 1990 and December 31, 2016. Demographic information of the patients is shown in Table 1. Healthy liver samples from organ donors were used as controls. Consistent with prior reports (28, 38), NRP1 and NRP2 expression was limited to LSECs in healthy liver; hepatocytes did not display specific immunoreactivity (Figures 1A–D). All HBs exhibited NRP1 expression; in 10/20 staining intensity was low or intermediate, and 10/20 samples had high NRP1 expression (Figures 1E,F,I,J, Table 1). NRP2 expression was observed in 19/20 HBs; it was low or intermediate in 11/20 and high in 8/20 samples (Figures 1G,H,K,L, Table 1). HB cells exhibited both cytoplasmic and membranous staining for NRP1 and NRP2. Additionally, LSCEs and hepatic stellate cells showed high NRP1 and NRP2 immunoreactivity in HB tissue (Figures 1G–L).

**Table 1:** Demographic variables and NRP expression in HBs.

**Variable**	**Number of HB patients (*n* = 20)**	**% of total**
**Sex**		
Female	10	50%
Male	10	50%
**Age at time of surgery (y)**		
<5	16	80%
>5	4	20%
**NRP1 expression**		
Negative	0	0%
Low/intermediate	10	50%
High	10	50%
**NRP2 expression**		
Negative	1	5%
Low/intermediate	11	55%
High	8	40%

**Figure 2:**
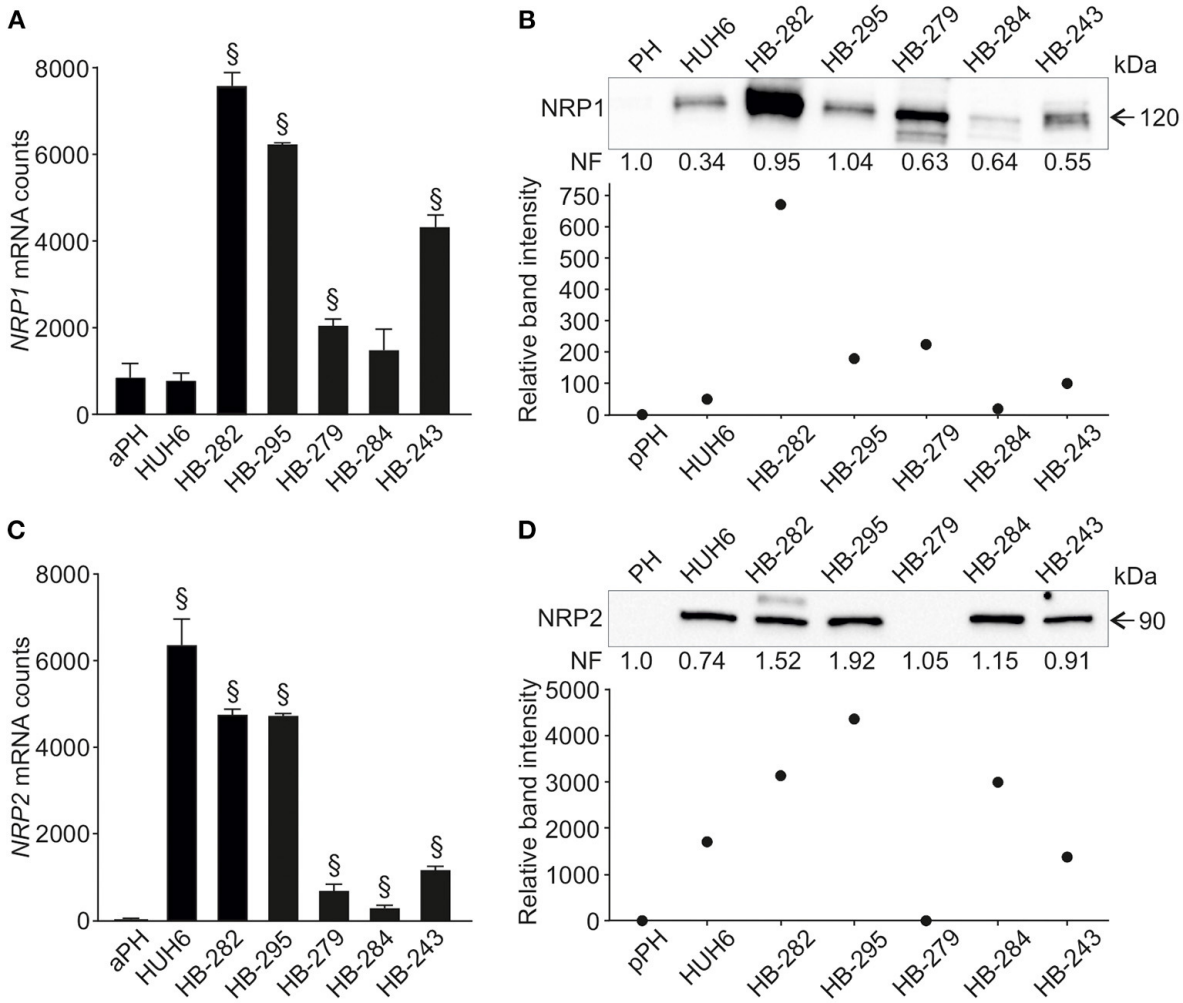
NRP1 and NRP2 RNA and protein expression in HB cell lines. *NRP1* mRNA expression was significantly higher in 4/6 HB cell lines investigated compared to adult primary hepatocyte control **(A)**. Similarly, NRP1 protein expression was elevated in same four cell lines **(B)** compared to pediatric primary hepatocytes. Upregulated NRP2 expression was observed in 5/6 HB cell lines both at mRNA **(C)** and protein level **(D)**. ^§^Adjusted *p*-value < 0.05. aPH, adult primary hepatocytes; pPH, pediatric primary hepatocytes. Band intensity is normalized to total protein expression of each lane. Normalization factor (NF) describing the amount of total protein in lane in relation to other lanes is given beneath the bands **(B,D)**.

Next, we evaluated NRP1 and NRP2 expression in six human HB cell lines and primary hepatocyte cultures. *NRP1* mRNA was upregulated in four HB cell lines (HB-282, HB-295, HB-279, HB-243) compared to primary hepatocytes (Figure 2A). Similarly, these four cell lines demonstrated a 70- to 700-fold increase in NRP1 protein levels compared to primary hepatocytes (Figure 2B). Upregulation of *NRP2* mRNA was noted in all HB cell lines while expression in primary hepatocytes was barely detectable (Figure 2C). At the protein level, HUH6, HB-282, HB-295, HB-284, and HB-243 demonstrated striking amounts of NRP2 (1,400- to 4,300-fold increase compared to primary hepatocytes; Figure 2D). NRP2 protein expression was undetectable in primary hepatocytes and HB-279 cells (Figure 2D).

### NRP2 Silencing in HUH6 and HB-282 Cell Lines

**Figure 3:**
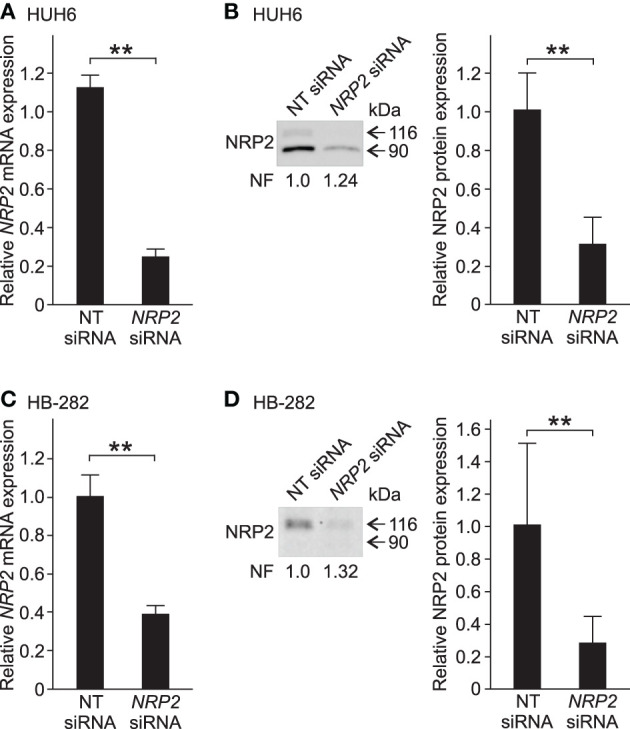
*NRP2* silencing in HB cells. In HUH6 cells, *NRP2* mRNA expression was reduced 75% after 72 h of *NRP2* siRNA transfection compared to NT control **(A)**. Protein band intensity of NRP2 was 70% lower in *NRP2* siRNA treated cells in contrast to NT control cells **(B)**. In HB-282 cells, NRP2 expression was reduced 60% at mRNA and 70% at protein level in *NRP2* siRNA transfected cells compared to control cells **(C,D)**. Bar plots are presented as relative values of mean of three independent experiments ± RSD. Band intensity is normalized to total protein expression of each lane. Normalization factor (NF) describing the amount of total protein in lane in relation to other lanes is given beneath the bands **(B)**. **p-value < 0.01. NT, non-targeting.

Since NRP2 expression was prominent in five out of six HB cell models, it was selected as the target for functional studies. We performed *NRP2* knockdown in HUH6 and HB-282 cells, which displayed the highest *NRP2* mRNA expression. To confirm the performance of the chosen transfection methods, HUH6 and HB-282 cells were transfected with a transfection indicator. Majority of the cells demonstrated sufficient intake of siGLO Green (Supplementary Figures 2A–F). Following transient siRNA transfections, NRP2 expression was reduced 70–75% at the mRNA and protein level in HUH6 cells (Figures 3A,B) and 60–70% at mRNA and protein level in HB-282 cells (Figures 3C,D).

### Knockdown of NRP2 Attenuates HB Cell Viability

**Figure 4:**
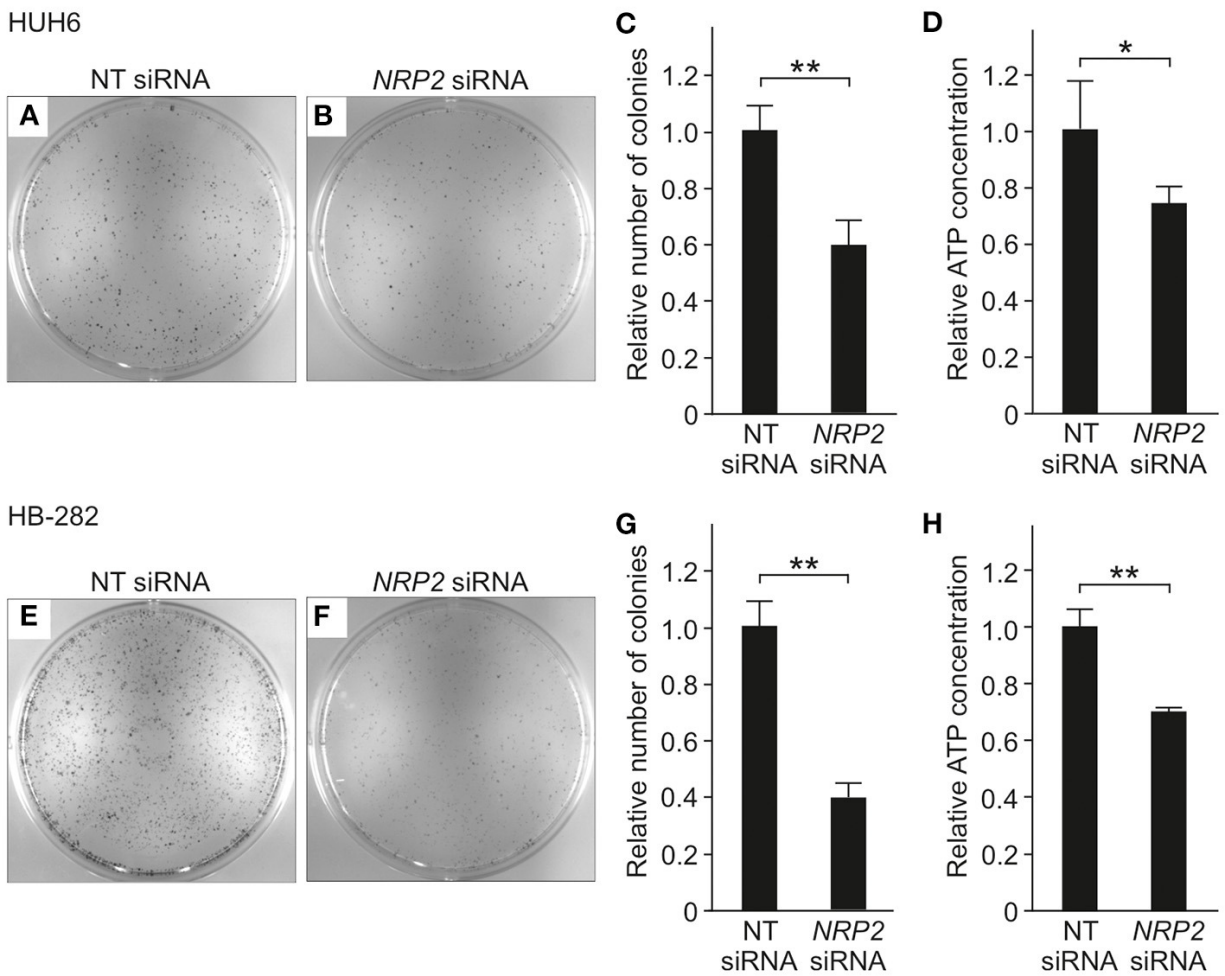
Viability in HB cells transfected with NT or *NRP2* siRNA. The number of colonies was decreased ~40% after *NRP2* knockdown in HUH6 cells **(A–C)** and 60% in HB-282 cells at 72 h post-transfection **(E–G)**. ATP concentration decreased 30% after *NRP2* silencing both in HUH6 **(D)** and HB-282 cells **(H)**. Bar plots are presented as relative values of mean of three independent experiments ± RSD. **p-value < 0.01, *p-value < 0.05. NT, non-targeting.

To evaluate the impact of *NRP2* silencing on cell growth and survival, we performed a clonogenic assay. A statistically significant reduction in the number of colonies was observed in both HUH6 (Figures 4A–C) and HB-282 (Figures 4E–G) cell lines, the decrease in colony numbers being 40 and 60%, respectively. The effect of *NRP2* knockdown on ATP availability was assessed as a secondary measure for cell viability. Approximately a 30% decrease in ATP concentration was noted in HUH6 (Figure 4D) and HB-282 (Figure 4H) cells.

### NRP2 Silencing Decreases Stress Fiber Formation and Attenuates HB Cell Motility

**Figure 5:**
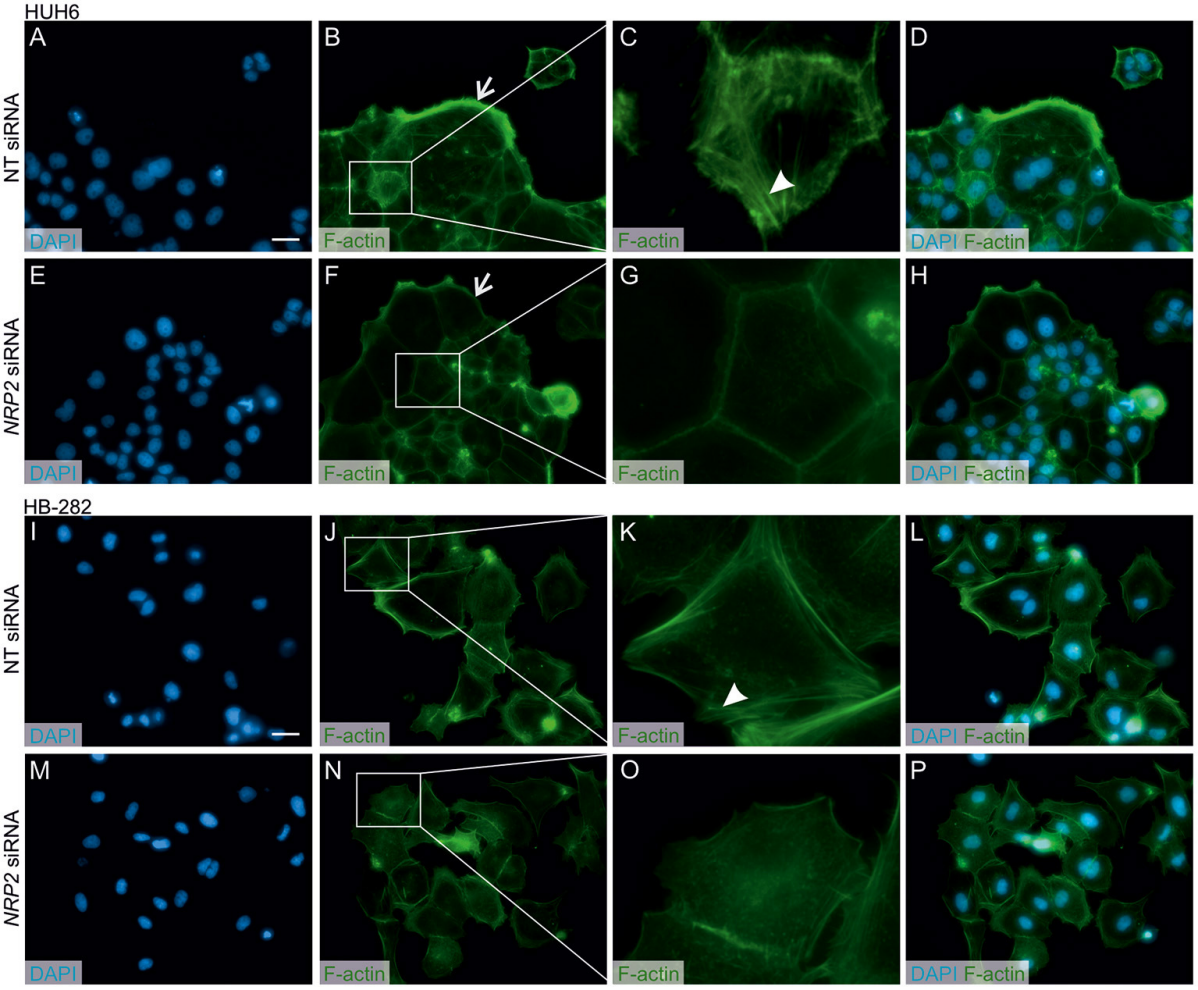
*NRP2* silencing reduces stress fiber formation and actin protrusions in HB cells. Nuclear staining with DAPI in NT **(A,I)** or *NRP2* siRNA **(E,M)** treated cells. Cells having undisturbed *NRP2* expression **(B,J)** stained with F-actin demonstrated more prominent cellular protrusions (arrow) and cytoplasmic accumulation of stress fibers compared to *NRP2* knockdown cells **(F,N)**. Close-up images **(C,G,K,O)** of **(B,J)** and **(F,N)** showing localization of stress fibers (arrowhead). Merged images of DAPI and F-actin staining in NT control cells **(D,L)** and *NRP2* knockdown cells **(H,P)**. Scale bar: 20 μm **(A,B,D–F,H–J,L–N,P)**. NT, non-targeting.

**Figure 6:**
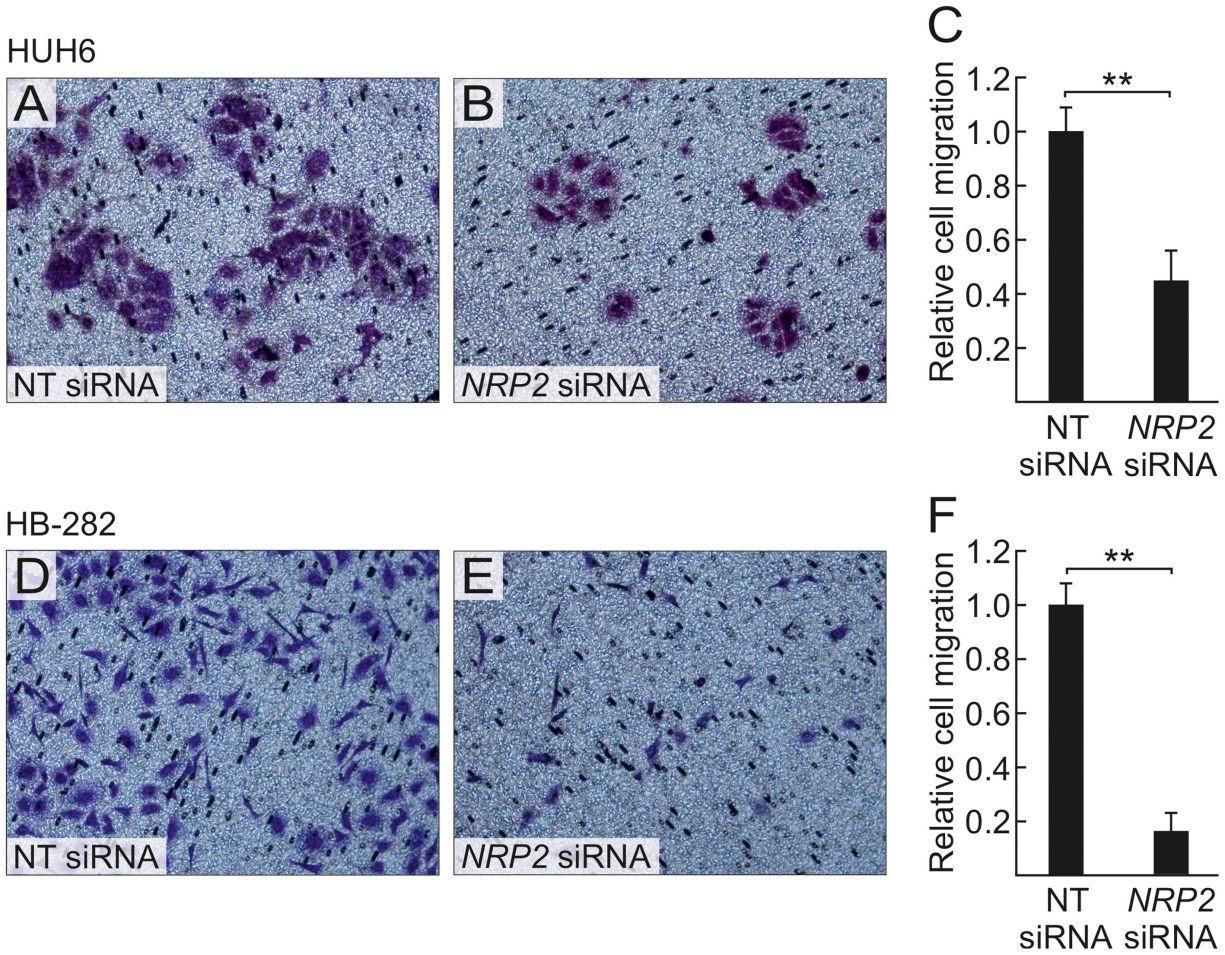
*NRP2* silencing decreases HB cell migration. After 40 h, decreased numbers of *NRP2* siRNA treated HUH6 and HB-282 cells migrated through the transwell membrane when compared to cells with an intact *NRP2* expression **(A,B,D,E)**; the relative cell migration was 2.2-fold higher in HUH6 and 6.2-fold higher in HB-282 NT control cells compared to *NRP2* silenced cells **(C,F)**. Bar plots are presented as relative values of mean of three independent experiments ± RSD. **p-value < 0.01. NT, non-targeting.

A previous study by Wittmann and colleagues demonstrated that NRP2 expression is associated with increased motility of liver carcinoma cells (28). We observed a reduced amount of actin protrusions and depolymerization of stress fibers in *NRP2* siRNA treated HUH6 (Figures 5E–H) and HB-282 (Figures 5M–P) cells compared to NT siRNA treated control cells (Figures 5A–D,I–L). Next, we investigated the motility of HUH6 and HB-282 cells exploiting the transwell migration assay. A statistically significant 55% (HUH6) and 85% (HB-282) decrease in the number of migrated cells was noted in HB cells with downregulated *NRP2* expression cells (Figures 6B,C,E,F) compared to control cells (Figures 6A,C,D,F).

## Discussion

**Figure 7:**
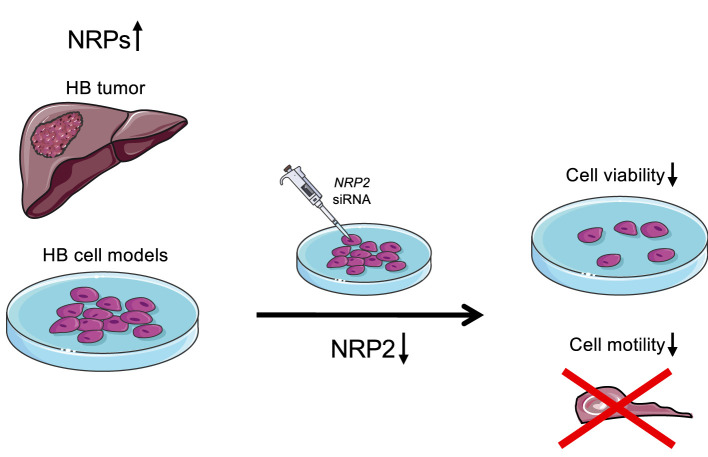
Schematic illustration of the findings. Majority of HB patient samples demonstrated high NRP1 and/or NRP2 expression. Furthermore, NRP2 silencing led to decreased cell viability and motility in HUH6 HB cell line.

NRPs have been associated with increased malignant potential and poor prognosis in various human cancers (19, 22, 39–41). In addition to their potential as biomarkers, efforts are underway to develop NRP1 or NRP2 as therapeutic targets (42–45). To the best of our knowledge, the present study is the first to demonstrate that NRP1 and NRP2 are highly expressed in HB. Furthermore, our observations link NRP2 expression with increased cell survival, actin stress fiber polymerization, and migration of HB cells (Figure 7). Currently, chemotherapy with cisplatin and doxorubicin is one of the mainstays of HB therapy, but multiorgan toxicity and chemoresistance limit the usability of these agents (46–49). Therefore, there is a need to identify tumor specific proteins to enable the development of targeted treatments for HB.

In adults with hepatocellular carcinoma, NRP2 expression correlates with shorter disease-free survival and overall survival (19). Moreover, high NRP2 expression was noted in de-differentiated tumors and mesenchymal hepatocellular carcinoma cell lines (19, 28). We observed high NRP2 expression in majority of HB tumors and *in vitro* models. Taken together, it appears that NRP2 expression is a common feature in poorly differentiated hepatic malignancies. Due to the limited number of patient samples, NRP2 expression was not correlated with clinical variables in this study. Therefore, in future investigations with larger sample sets, the association of NRP2 with HB patient survival should be explored.

NRP2 expression has been associated both with increased proliferation rate as well as with the capability of tumor cells to escape from programmed cell death in cancer cells (13, 50, 51). We observed decreased cell viability in *NRP2* knockdown cells, but further studies are needed to clarify whether this is a consequence of lower proliferation rate or increased cell death. Interestingly, a recent study linked upregulation of *NRP2* with cisplatin-resistance in neuroblastoma cells (52). Poor response to current chemotherapeutical treatments, including cisplatin, have been observed in 20–25% of HB tumors pointing out the need to find approaches to overcome the chemoresistance (49). It would be of interest to study whether cisplatin treatment combined with NRP2 targeting could cause an even more drastic decrease in cell viability in HB cells than cisplatin administration or *NRP2* knockdown alone.

Actin stress fibers are a hallmark of mesenchymal phenotype, and remodeling of actin cytoskeleton is a prerequisite for cancer cell motility (53). Polymerized stress fibers form protrusive structures named filipodia, lamellipodia, and invadopodia directing the migration and invasion of cancer cells to new locations (54). This process is a preliminary step in metastasis formation (55). High NRP2 expression has been associated with increased motility and invasiveness in cancer cell models (28, 56, 57). In line with these findings, *NRP2* silencing led to depolymerization of actin stress fibers, and consequently decreased the migration of HB cells. The importance of NRP2 in pre-metastatic processes suggests that NRP2 targeted interventions hold potential in the management of aggressive HB.

A multitude of signaling mediators are known to interact with NRPs. In breast cancer cells, neutralizing NRP2 antibody blocked cytoplasmic C-X-C Motif Chemokine Receptor 4 (*CXCR4*) expression which was followed by decreased tumor cell migration (22). Interestingly, upregulation of CXCR4 was recently associated with the infiltration of pro-tumorigenic macrophages, neutrophils, and dendritic cells in HB tissue samples (58). Considering the NRP2-CXCR4 axis, NRP2 targeting might attenuate the growth of HB at multiple levels. In addition to the direct effect on HB cells observed in our study, anti-NRP2 approaches might disrupt the tumor microenvironment.

Abnormal WNT/β-catenin signaling is observed in the majority of HBs, and several studies have shown that inhibition of WNT/β-catenin suppresses HB cell growth *in vitro* (7, 59–61). Captivatingly, upregulation of secreted WNT antagonists decreased NRP2 expression in osteosarcoma cell models suggesting that NRP2 transcription is regulated by WNT pathway (44). NRP2 expression has also been directly connected to improved β-catenin stability with consequently increased motility and invasiveness of gastrointestinal cancer cells (57). Future investigations should explore whether upregulation of NRP2 in HBs is linked to aberrant WNT/β-catenin signaling activity.

Major limitations of this study were the rather low number of patient samples and challenges linked to the siRNA mediated silencing method. It is known that siRNAs may have off-target effects (62). Therefore, it should be noted that the *in vitro* results presented in this study may not be explicitly due to the *NRP2* silencing. In the future studies, another silencing methods, such as CRISPR-Cas9 mediated gene editing, should be considered to overcome this issue.

In conclusion, NRPs are expressed in the majority of HBs and further studies are warranted to evaluate their potential as prognostic biomarkers. Moreover, RNA interference mediated inhibition of NRP2 suppresses HB cell viability and motility suggesting that NRP2 targeted interventions have potential in the management of aggressive HB.

## Data Availability Statement

Publicly available datasets were analyzed in this study. This data can be found here: https://www.ncbi.nlm.nih.gov/geo/ (accession numbers: GSE83518 and GSE140520) and https://ega-archive.org/datasets (Study ID: EGAS00001004827, dataset EGAD00001006621).

## Ethics Statement

The studies involving human participants were reviewed and approved by Helsinki University Hospital institutional ethics committee. Written informed consent to participate in this study was provided by the participants' legal guardian/next of kin.

## Author Contributions

KE, MP, and MH: conceptualization and research design. KE, RN, MPP, DW, and MP: acquisition, analysis, or interpretation of data. SC: establishing and providing PDX cell models. MP: preparing the final figures. KE: writing the first draft. KE, RN, SC, MPP, DW, MP, and MH: reviewing and editing and final approval of the manuscript version to be published. All authors contributed to the article and approved the submitted version.

## Conflict of Interest

SC is employed by the company XenTech. The remaining authors declare that the research was conducted in the absence of any commercial or financial relationships that could be construed as a potential conflict of interest.

## Abbreviations

CXCR4: C-X-C chemokine receptor type 4
FFPE: formalin-fixed paraffin-embedded
GAPDH: glyceraldehyde 3-phosphate dehydrogenase
HB: hepatoblastoma
HSC: hepatic stellate cell
LSEC: liver sinusoidal endothelial cell
NRP1: neuropilin-1
NRP2: neuropilin-2
NT: non-targeting
PDX: patient-derived xenograft
PPIG: peptidyl-prolyl cis-trans isomerase G
siRNA: small interfering RNA.
