# Supplementary material for: Neuropilin-2 Is Associated With Increased Hepatoblastoma Cell Viability and Motility
Source: Front Pediatr. 2021 Jun 22;9:660482. doi: 10.3389/fped.2021.660482 (PMC8257959; doi:10.3389/fped.2021.660482)
Supplement: Supplementary file 1 [file Data_Sheet_1.PDF]

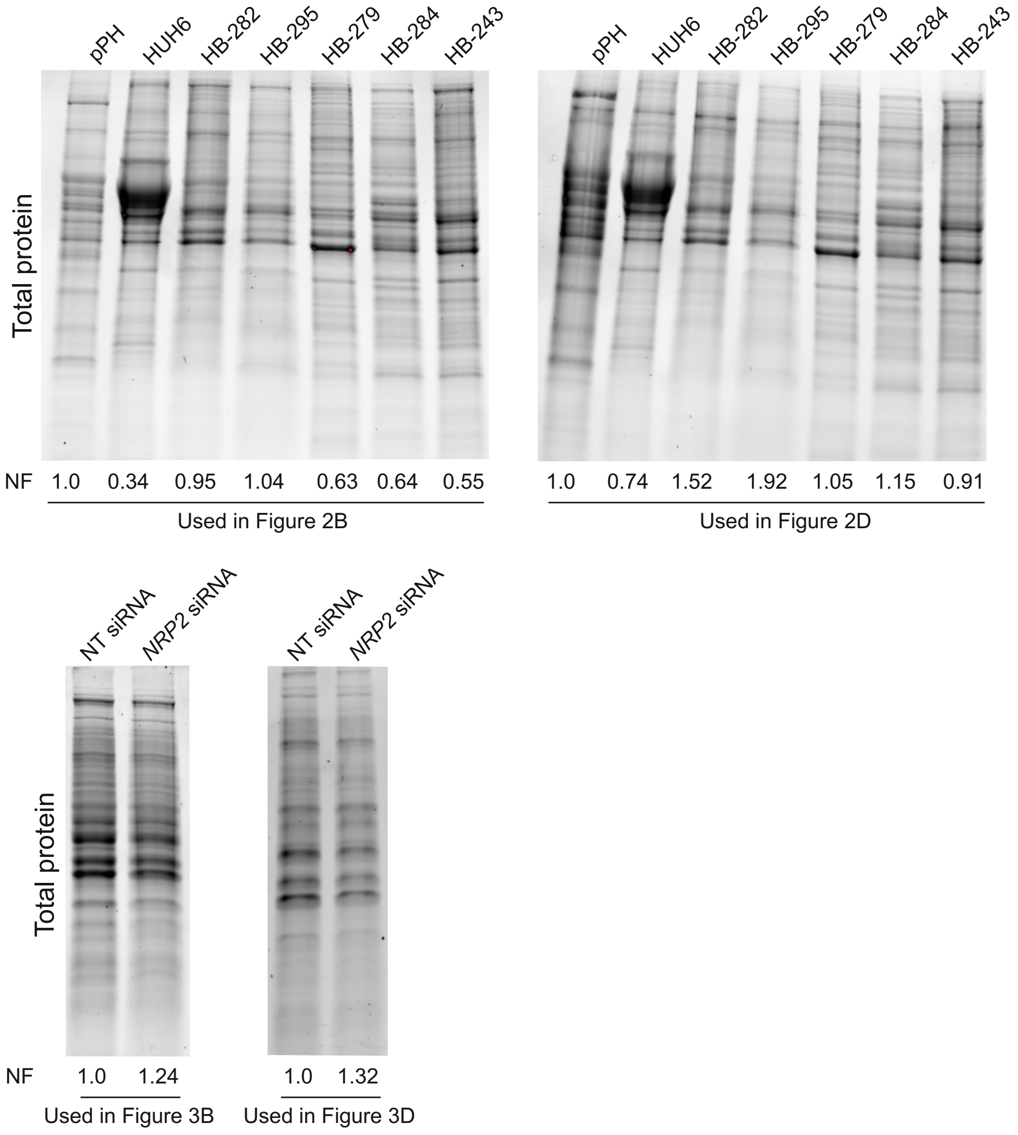

Supplementary figure 1. Total proteins detected by Stain-Free technology. Normalization factor (NF) describes the amount of total protein in lane in relation to other lanes. NF = normalization factor.
