# Supplementary material for: Neuropilin-2 Is Associated With Increased Hepatoblastoma Cell Viability and Motility
Source: Front Pediatr. 2021 Jun 22;9:660482. doi: 10.3389/fped.2021.660482 (PMC8257959; doi:10.3389/fped.2021.660482)
Supplement: Supplementary file 2 [file Data_Sheet_2.PDF]

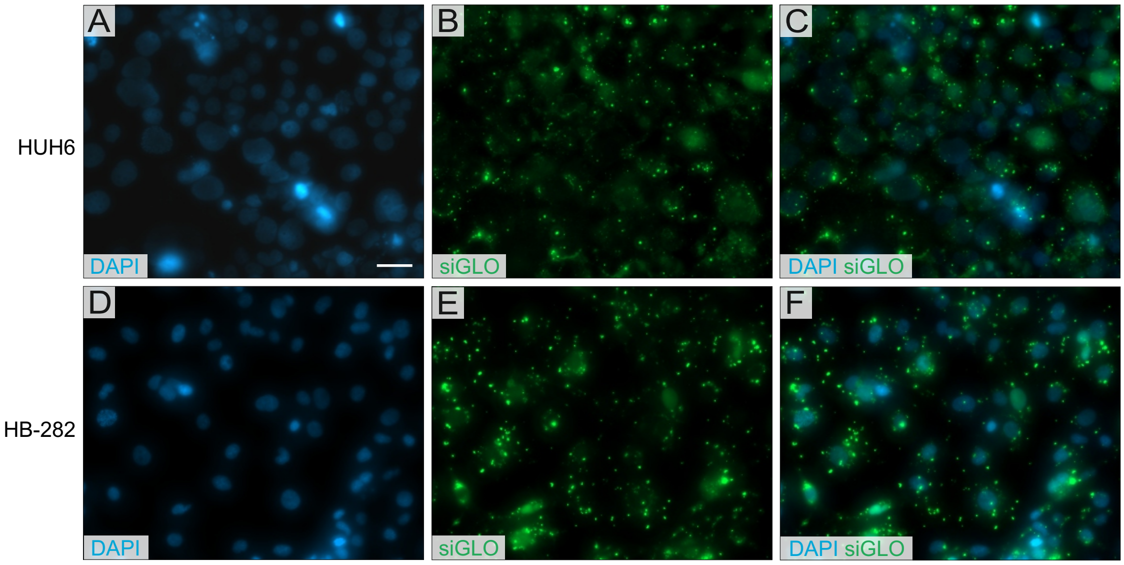

Supplementary figure 2. Assessment of transfection efficacy using siGLO Green transfection indicator in HUH6 (A-C) and HB-282 (D-F) cells at 24 h post-transfection. Scale bar = 20µm.
